# Functionalized Cyclodextrin/Carboxymethyl Cellulose Composite Hydrogel with Double Network Structure for Adsorption of Heavy Metal Ions in Wastewater

**DOI:** 10.3390/molecules29225414

**Published:** 2024-11-16

**Authors:** Hong Zhang, Xiaodong Yang, Xin Zhang, Wenbin Liu, Meiqing Fan, Lei Wang

**Affiliations:** 1 Jilin Provincial Key Laboratory of Straw-Based Functional Materials, Institute for Interdisciplinary Biomass Functional Materials Studies, Jilin Engineering Normal University, Changchun 130052, China; zhanghong0825@jlenu.edu.cn (H.Z.); yangxd@jlenu.edu.cn (X.Y.); zhangxin0422@jlenu.edu.cn (X.Z.); 2 Key Laboratory of Superlight Materials and Surface Technology, Ministry of Education, College of Materials Science and Chemical Engineering, Harbin Engineering University, Harbin 150009, China; liuwenbin@hrbeu.edu.cn; 3 Key Laboratory of Molecular Enzymology and Engineering of Ministry of Education, Jilin University, Changchun 130023, China

**Keywords:** functionalized cyclodextrin/carboxymethyl cellulose hydrogels, heavy metal ions, adsorption isotherm, adsorption kinetics

## Abstract

Heavy metal ions in industrial wastewater pose significant environmental and ecological threats. In this work, a hydrogel featuring a double network structure was synthesized via radical polymerization and cross-linking of β-cyclodextrin (CD) and carboxymethylcellulose (CMC) with acrylic acid (AA). The hydrogel’s functional groups and microstructure were characterized using Fourier transform infrared spectroscopy (FTIR-ATR), X-ray diffraction (XRD), scanning electron microscopy (SEM), and thermogravimetric analysis (TGA). Mechanical properties were evaluated through rheological and compression tests. The study examined the impact of initial metal ion concentration, adsorbent-ion contact time, and solution pH on adsorption capacity. The maximum adsorption capacities of the functionalized CD/CMC-PAA-MBA hydrogel for Cu^2+^, Pb^2+^, and Cd^2+^ ions were 158.12, 393.56, and 290.12 mg/g, respectively. Notably, the hydrogel exhibited the highest selectivity for Pb^2+^ in mixed solutions. The adsorption kinetics of the metal ions were modeled using the pseudo-second-order rate equation and the Langmuir adsorption isotherm.

## 1. Introduction

The global industrial and manufacturing sectors have experienced unprecedented growth, leading to a corresponding surge in wastewater discharges. This effluent often contains contaminants such as heavy metals, dyes, and organic substances, which, if left untreated, pose significant threats to precious freshwater resources, ecosystems, and human and animal health [1,2]. Heavy metal ions, typically formed by the loss of electrons from metals with densities greater than 4.5 g/cm^3^ or atomic masses exceeding 55, are notorious for their non-biodegradability, toxicity, and carcinogenic potential. When released into the environment, these ions degrade the quality of drinking water and necessitate urgent removal mechanisms [3,4,5]. Current methods for wastewater treatment include chemical, physical, and bioprocesses, with the adsorption method—known for its low production costs, widespread availability of raw materials, high economic recycling rate, and efficiency in removing heavy metals—emerging as a superior option [6,7,8,9]. Given the widespread application of adsorption in heavy metal treatment, there is a pressing need to develop efficient, bio-based adsorbents for heavy metal removal in water.

Hydrogels, characterized by their three-dimensional, cross-linked polymer structures and the ability to absorb substantial amounts of water without dissolving, are renowned for their exceptional water absorption, high porosity, diverse forms, and ease of post-processing. They boast a significant advantage over other adsorbent materials in the removal of heavy metal ions, garnering substantial scientific interest due to this capability [10,11,12].

In recent years, researchers have increasingly focused on biomass materials as a promising renewable resource. Among these, natural cellulose stands out as the most abundant polysaccharide in nature, widely distributed and commonly utilized as a green material [13]. However, the inherent insolubility of cellulose poses challenges for value-added applications. Through chemical modification, carboxymethylcellulose (CMC) can be synthesized, offering enhanced solubility and applicability across medicine, food, environmental protection, and agriculture. Despite its versatility, standalone carboxymethyl cellulose hydrogel exhibits limitations such as brittleness, low mechanical strength, and poor mechanical performance. These shortcomings can be mitigated through chemical modification, particularly through blending with other polymers, graft copolymerization, or ether system modifications, with graft copolymerization being the most widely adopted method for improving heavy metal ion adsorption [14,15,16,17].

β-Cyclodextrin, a macrocyclic compound comprising seven glucose units derived from starch hydrolysis, features a hydrophobic cavity capable of encapsulating organic substances, forming inclusion complexes. The external hydroxyl groups on the cyclodextrin surface act as active reaction sites, enabling interactions with a broad array of functional groups and compounds through van der Waals forces and hydrophobic interactions, including organic pollutants and metal ions [18,19,20]. Given its unique structural characteristics and the combined properties of cyclodextrin and other molecules, it has emerged as a focal point for studies on pollutant removal, particularly as an adsorbent.

**Figure 1:**
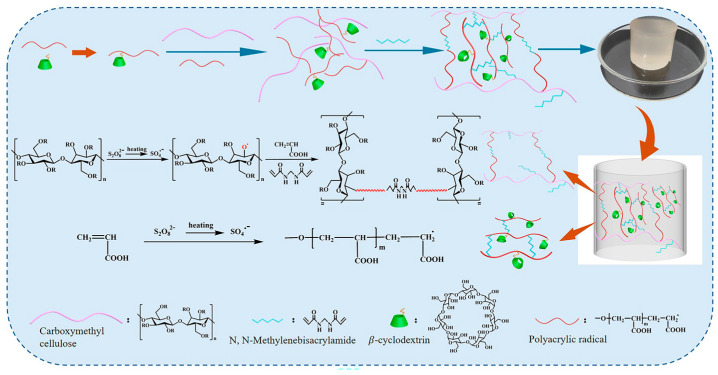
Synthesis process of composite hydrogel CD/CMC-PAA-MBA.

This study uses carboxymethyl cellulose (CMC) and β-cyclodextrin as substrates, separately reacting with ammonium persulfate (APS) to effect grafting of the flexible long-chain polymer, carboxymethyl cellulose (CMC), via a free radical polymerization process. The outer hydroxyl groups of β-cyclodextrin (CD) were functionalized with the cross-linking agent *N*,*N*′-methylenebisacrylamide (MBA) to establish a robust network structure, yielding a stable double-network hydrogel material stabilized via hydrogen bonding and electrostatic interactions (Figure 1). The internal architecture of the carboxymethyl cellulose (CMC) exhibited numerous capillaries and a substantial specific surface area, conferring significant advantages for the adsorption of heavy metal ions [21]. The structural and morphological features of the material were meticulously characterized using Fourier transform infrared spectroscopy (FTIR-ATR), scanning electron microscopy (SEM), X-ray diffraction (XRD), and thermogravimetric analysis (TGA). The mechanical properties were evaluated through rheological and compression testing. Furthermore, the influence of parameters such as pH, concentration, and contact time of the metal ion solution on adsorption was systematically investigated. The adsorption capacity of the composite hydrogel materials towards metal ions was assessed using adsorption isotherms and kinetics, and the reusability of the hydrogels was explored.

## 2. Results and Discussion

### 2.1. Structural Characterization and Analysis of CD/CMC-PAA-MBA

#### 2.1.1. FTIR-ATR Analysis

**Figure 2:**
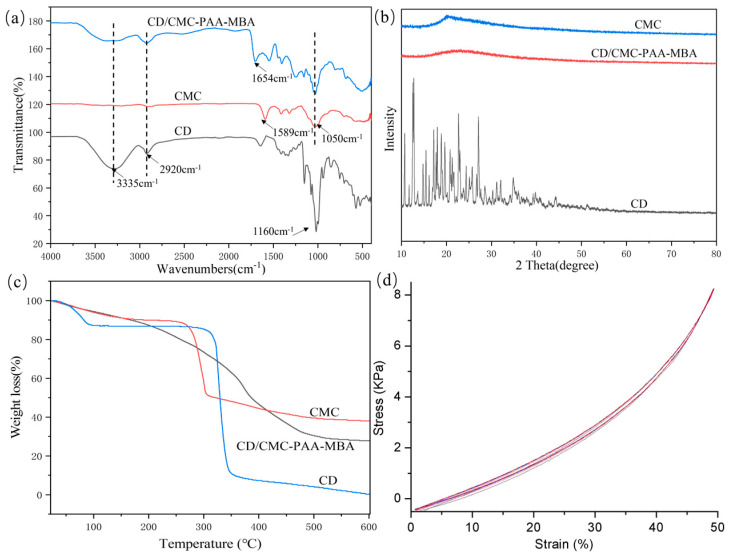
(**a**) FTIR-ATR spectra of CD, CMC, and CD/CMC-PAA-MBA; (**b**) XRD curves of CD, CMC, and CD/CMC-PAA-MBA; (**c**) TGA curves of CD, CMC, and CD/CMC-PAA-MBA; (**d**) 10 cycles of compression curves of CD/CMC-PAA-MBA.

The functional groups in the modified β-CD/CMC hydrogels were identified using FTIR-ATR spectroscopy, as illustrated in Figure 2a. The CMC spectrum displayed characteristic peaks typical of carboxymethyl cellulose. Notably, the absorption band near 1589 cm^−1^ corresponded to the COO^−^ stretching vibration, while the band near 3354 cm^−1^ was associated with O-H groups. The sharp peak at 2903 cm^−1^ resulted from C-H stretching vibration, and the absorption peaks at 1430 cm^−1^ and 1050 cm^−1^ were indicative of -CH_2_ groups and C-O-C in glucose [22]. The CD spectrum featured a broad absorption band at 3335 cm^−1^, attributable to the stretching vibration of -OH. Additionally, a sharp IR absorption band at 2920 cm^−1^ indicated the stretching vibration of -CH-. The characteristic peak at 1160 cm^−1^ in the IR spectrum corresponded to the cyclic sugar ring C-C structure of the glucose molecule in cyclodextrin [23]. In the CD/CMC-PAA-MBA spectra, the fundamental structures of β-CD and CMC were preserved, and a new peak at 1654 cm^−1^ emerged, signifying the stretching vibration of the C-N bond. These observations confirmed the successful incorporation of AA and MBA into CD and CMC via radical graft copolymerization.

#### 2.1.2. X-Ray Diffraction Analysis

The crystal structures of CMC, CD, and CD/CMC-PAA-MBA hydrogels were examined using X-ray diffraction (XRD), with the results presented in Figure 2b. β–CD exhibited distinct characteristic diffraction peaks at 10–27°, consistent with the β–CD standard from the PDF card (JCPDS No. 47–2421). The CMC spectrum revealed a broad, strong diffraction peak at 20.23°, indicating a degree of crystallinity. Conversely, the prepared CD/CMC–PAA–MBA hydrogel showed only weak diffraction peaks between 18.02 and 27.85°. These observations suggest that graft polymerization enhanced the amorphous nature of the polymers, transitioning them from crystalline to amorphous states [24]. The consistency of these findings with previous FTIR-ATR analyses confirms the successful grafting of AA and MBA onto CD and CMC.

#### 2.1.3. Thermogravimetric Analysis

The thermal stability and decomposition behavior of CD/CMC-PAA-MBA hydrogels were analyzed via TGA, with the results depicted in Figure 2c. The decomposition of the composite hydrogel was primarily partitioned into three distinct stages. The initial stage, occurring in the range 50–200 °C, exhibited a weight loss of 12.2%, attributable to the evaporation of physically bound water within the β-CD cavity. The second stage, spanning 210–300 °C, showed an 18.2% mass loss, primarily due to the thermal degradation of β-CD, CMC macromolecular chains, PAA grafts, and MBA. The third stage, defined by a sharp decomposition between 310 and 450 °C, resulted in a 30.2% mass loss, largely attributed to the thermal decomposition or vaporization of organic small molecules and residual byproducts [25]. These findings underscore the robust thermal stability of the CD/CMC-PAA-MBA composite hydrogel.

#### 2.1.4. Compression Properties Analysis

During the adsorption of heavy metal ions, the hydrogel must exhibit adequate mechanical strength. To assess this property, cyclic compression tests were conducted on the prepared hydrogels. As depicted in Figure 2d, the hydrogel exhibited a minimal area of hysteresis in ten consecutive compression cycles, with consistent hysteresis areas each time. This behavior indicates a rapid recovery process, supported by the elastic modulus and hysteresis loop calculations presented in Figure 2d. Within a certain range of compressive deformation, the compression was reversible. This suggests the presence of strong intermolecular forces within the designed hydrogel, conferring it reversible and rapid compressive recovery characteristics. Consequently, the hydrogel maintains structural integrity even after adsorbing significant amounts of heavy metal ions, facilitating its recovery and reuse in subsequent experiments [26].

#### 2.1.5. Rheological Properties Analysis

**Figure 3:**
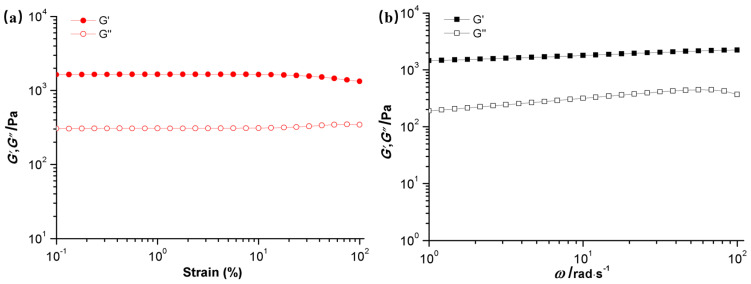
(**a**) Storage modulus (G′) and loss modulus (G″) vs strain of the CD/CMC−PAA−MBA hydrogels and (**b**) storage modulus (G′) and loss modulus (G″) vs angular frequency of the CD/CMC-PAA−MBA hydrogels.

The rheological properties of this hydrogel series were analyzed, focusing on the relationship between viscoelasticity and mechanical strength. Specifically, the storage modulus (G′) and loss modulus (G″) were examined under oscillatory frequency and strain scanning modes. As depicted in Figure 3a, the hydrogels’ G′ values were consistently higher than their G″ values across the frequency range of 1–100 rad/s, indicating the formation of stable hydrogels. The CD/CMC-PAA-MBA hydrogel, for instance, exhibited an average storage modulus of 2077.45 Pa and a loss modulus of approximately 198.04 Pa, with a significant difference of 1879.41 Pa. This substantial disparity suggests that the CD/CMC-PAA-MBA hydrogel possesses excellent elastic properties and consistent mechanical strength, which aligns with its ability to maintain its structural integrity during metal ion adsorption. Furthermore, as shown in Figure 3b, the G′ and G″ of the hydrogels were assessed at a constant oscillatory frequency of 10 rad/s across a strain range of 0 to 100%. The results revealed that both G′ and G″ remained stable as the strain increased from 0 to 100%, with minimal differences between them. The small disparity in G′ and G″ indicates that these hydrogels exhibit stable and relatively robust mechanical performance and elasticity, which are advantageous for the efficient adsorption of metal ions [27].

#### 2.1.6. Surface Morphology Analysis

**Figure 4:**
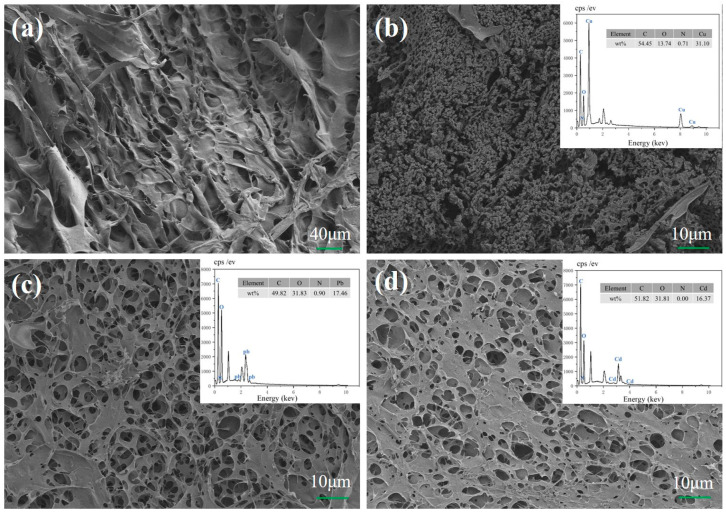
SEM images of (**a**) CD/CMC-PAA-MBA, (**b**) SEM images of Cu^2+^-loaded CD/CMC−PAA−MBA, (**c**) SEM images of Pb^2+^−loaded CD/CMC−PAA−MBA, and (**d**) SEM images of Cd^2+^−loaded CD/CMC−PAA−MBA.

The surface morphology and properties of CMC and β–CD composite hydrogels were investigated through SEM analysis, as depicted in Figure 4a. The observed surfaces were characterized by their rough, irregular, and uneven nature, featuring an evident porous structure with micropores of varying sizes. The internal architecture of the composite hydrogel revealed an intricate three-dimensional porous network, replete with numerous interconnecting microporous sheets and a thin, porous structure that exposed a multitude of active adsorption sites on the outer surface. This intricate network facilitated the diffusion of heavy metal ions into the hydrogel’s interior and their binding with the adsorption sites. Figure 4b–d present the SEM images of the composite hydrogels after loading with Cu^2+^, Pb^2+^, and Cd^2+^. The surfaces of these adsorbed hydrogels exhibit a significantly roughened appearance, with the inner walls of the pore channels hosting a dense array of tiny particles corresponding to the adsorbed Cu^2+^, Pb^2+^, and Cd^2+^ ions. This morphology suggests successful adsorption of these metal ions within the pores of the CD/CMC–PAA–MBA hydrogel, facilitated by the exposed lamellar surfaces. Furthermore, the elemental composition of the loaded hydrogels was examined using EDS analysis, as illustrated in the vignettes of Figure 4b–d. The presence of all three metal ions was detected in the hydrogel loaded with Cu^2+^, Pb^2+^, and Cd^2+^, unequivocally indicating their enrichment within the adsorbed sample.

### 2.2. Study of the Adsorption Properties of CD/CMC-PAA-MBA Hydrogels

#### 2.2.1. Effect of Solution pH on the Adsorption

**Figure 5:**
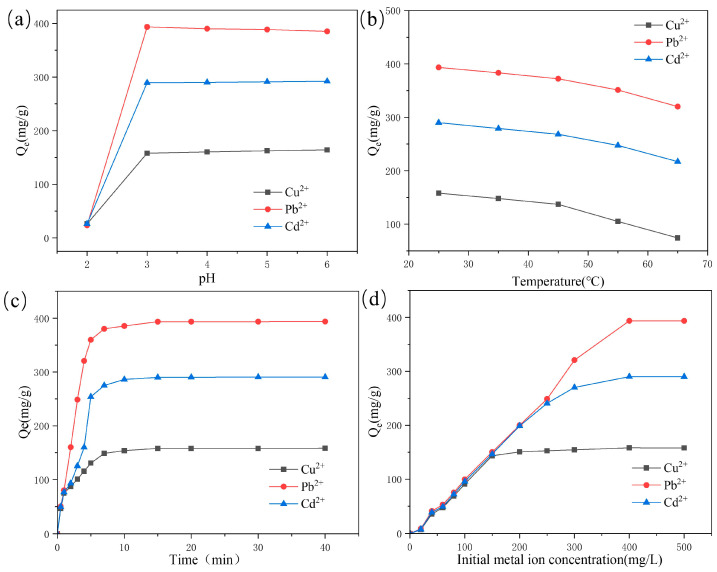
Effect of (**a**) pH, (**b**) temperature, (**c**) contact time, and (**d**) initial metal ion concentration on the adsorption of Cu^2+^, Pb^2+^, and Cd^2+^ ions by CD/CMC-PAA-MBA.

The pH of the solution affected the chelating copolymerization coordination process of heavy metal ions with hydrogel materials; therefore, the pH of the solution played a very important role in the adsorption properties of the hydrogel materials (Figure 5a). To analyze the effect of pH on adsorption efficiency, the isoelectric point (pH0) of the hydrogel was measured. At pH = 0, almost all surface groups of the adsorbent are protonated and carry a positive charge. Consequently, if the pH value is greater than pH0, the surface of the adsorbent will accumulate a negative charge, which is beneficial for the adsorption of metal ions [28]. In the present study, the variation of adsorption of Cu^2+^, Pb^2+^ and Cd^2+^ by adsorbent was investigated in the range of pH 2–5. The pH range of 2–5 was chosen because when the solution pH was 6, the state of the metal ions in the solution changed, leading to the appearance of precipitated hydroxides and flocs, which affected the adsorption properties of the hydrogel material, whereas at a pH less than 2, the concentration of H^+^ in the solution became higher, resulting in the surface of the hydrogel material being positively charged as well, and the phenomenon of repulsion of the same charge would occur, leading to a decrease in the adsorption capacity. The hydrogel exhibited substantial adsorption magnitude within the pH range of 2 to 5. Therefore, in order to overcome the occurrence of these two problems, the pH range of 2–5 was chosen as the optimal experimental condition [29].

#### 2.2.2. Effect of Temperature on the Adsorption

Temperature significantly influences the adsorption capacity of hydrogels, and its variation can elucidate whether the adsorption process is exothermic or endothermic. Figure 5b illustrates the adsorption capacities of the CD/CMC-PAA-MBA composite hydrogel for three metal ions (Cu^2+^, Pb^2+^, and Cd^2+^) across a temperature range of 25 to 65 °C. Notably, the adsorption capacity of the composite hydrogel for these metal ions progressively declined with increasing temperature. For instance, the adsorbed amount of Cu^2+^ decreased from 158.12 mg/g to 74.31 mg/g, Pb^2+^ from 393.56 mg/g to 320.17 mg/g, and Cd^2+^ from 290.12 mg/g to 217.18 mg/g as the temperature rose from 25 °C to 65 °C. This trend suggests that elevated temperature is detrimental to the adsorption capacity of the composite hydrogel. Consequently, the exothermic nature of the adsorption process indicates that lower temperatures, notably 25 °C, are optimal for achieving the highest adsorption efficiencies for these heavy metal ions.

#### 2.2.3. Effect of Contact Time on the Adsorption

The contact time between the hydrogel adsorbent and heavy metal ions significantly impacts adsorption performance. Figure 5c depicts the adsorption capacity of the CD/CMC-PAA-MBA composite hydrogel for three metal ions (Cu^2+^, Pb^2+^, and Cd^2+^) at varying contact times. As illustrated in Figure 5c, the composite hydrogel exhibited a dual-phase adsorption behavior, characterized by an initial rapid phase followed by a slower phase until saturation was achieved. Remarkably, within the first 5 min, the hydrogel rapidly adsorbed Cu^2+^, Pb^2+^, and Cd^2+^ to levels of 130.6 mg/g, 359.8 mg/g, and 253.9 mg/g, respectively. The adsorption equilibrium was attained within 15 min, primarily due to the saturation of binding sites on the adsorbent, which limited further capacity to bind additional metal ions. Notably, the adsorption time for this composite hydrogel, based on cellulose materials, was shorter than previously reported [30].

#### 2.2.4. Effect of Initial Metal Ion Concentration on the Adsorption

Figure 5d illustrates the impact of varying initial concentrations of Pb^2+^, Cu^2+^, and Cd^2+^ on the adsorption properties of the CD/CMC-PAA-MBA composite hydrogels. The data reveal that the adsorption capacity of the hydrogel increased substantially with higher initial concentrations of the metal ions, exhibiting a linear growth pattern at low concentrations and a plateau at higher concentrations. This behavior can be attributed to the fixed number of active adsorption sites within the hydrogel, which, under a constant amount of adsorbent, become progressively saturated as the metal ion concentration rises. Consequently, the hydrogel’s adsorption capacity initially escalates with increasing metal ion concentration but ultimately levels off, ceasing further chelation coordination with the heavy metal ions. The maximum adsorption capacities for Pb^2+^, Cu^2+^, and Cd^2+^ were determined to be 158.12 mg/g, 393.56 mg/g, and 290.12 mg/g, respectively.

#### 2.2.5. Adsorption Isotherm

In order to study the affinity between adsorbent and adsorbate, the difficulty of adsorption capacity, and the interrelationship between homogeneity and inhomogeneity on the adsorbent surface, the adsorption process of the prepared composite hydrogels was fitted and analyzed in this study using the Langmuir adsorption isothermal model and the Freundlich isothermal adsorption model, respectively. The Langmuir isothermal adsorption model is a classical monomolecular layer adsorption model for adsorbents whose surfaces are homogeneous and also illustrates that the interaction relationship between adsorbent and adsorbate is dominated by chemisorption (Equation (1)).The Freundlich isothermal adsorption model is a more ideal mathematical-empirical formula, which is mostly used in the case of materials whose surfaces are non-homogeneous and illustrates the interaction relationship between adsorbent and adsorbate is dominated by chemophysical adsorption (Equation (2)) [31].

Langmuir isothermal adsorption model:

(1) $$\frac{C_{e}}{Q_{e}}=\frac{1}{K_{L}Q_{m}}+\frac{C_{e}}{Q_{m}}$$

where Q_e_ is the adsorbed amount when the adsorbent reaches adsorption equilibrium in mg/g; Q_m_ is the theoretical maximum adsorption amount or saturation adsorption amount of the adsorbent in mg/g; K_L_ is the Langmuir’s constant in L/mg; and C_e_ is the concentration of the residual metal ions in the system when the adsorption by the adsorbent reaches equilibrium, in mg/L.

Freundlich isothermal adsorption model:

(2) $$lnQ_{e}=\frac{1}{n}lnC_{e}+lnK_{P}$$

Temkin isothermal adsorption model:

(3) $$Q_{e}=B\ln K_{T}+B\ln C_{e}$$

Q_e_ is the adsorbed amount when the adsorbent reaches adsorption equilibrium, in mg/g; C_e_ is the concentration of metal ions remaining in the system when the adsorption of the adsorbent reaches equilibrium, in mg/L; K_p_ is Freundlich’s constant; n is a characterization constant, and the range of values of the n-value is related to the degree of non-homogeneity of the surface of the adsorbent or the adsorption capacity of the adsorption sites on the adsorbent surface. That is, when n > 1 (or 0 < 1/n < 1), it indicates that the adsorption process of the adsorbent is occurs easily (this adsorption process can also be called preferential adsorption); when 0 < n < 1 (or 1/n > 1), it indicates that the adsorbed process of the adsorbent does not occur easily, where *K_T_* and *B* are the Temkin parameter related to the adsorption amount constants, respectively [32,33,34].

**Figure 6:**
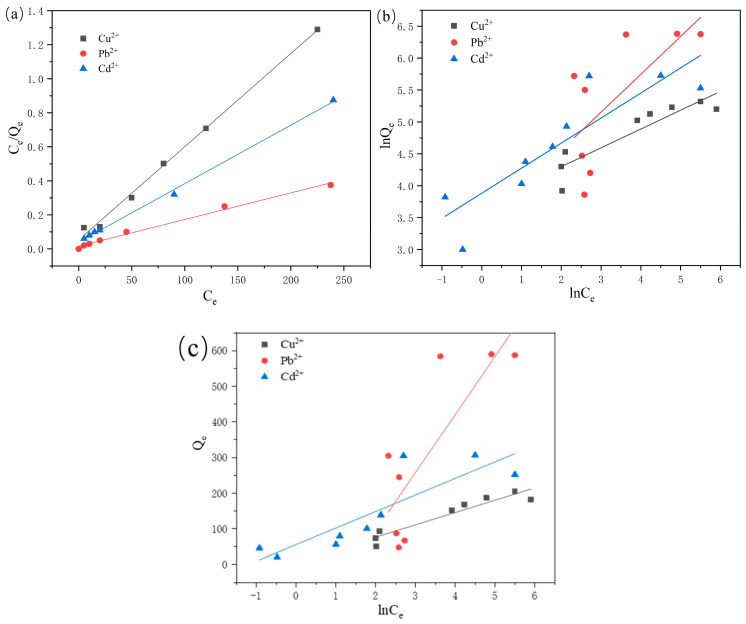
(**a**) Langmuir, (**b**) Freundlich, and (**c**) Temkin adsorption isotherm model for Pb^2+^, Cd^2+^, and Cu^2+^ adsorption.

In order to further investigate the adsorbed mechanism of the composite hydrogel on Pb^2+^, Cu^2+^, and Cd^2+^, as well as the difficulty of the adsorption process, the relevant experimental data were fitted via the Langmuir, Freundlich, and Temkin isothermal adsorption equations, respectively; the results are shown in Figure 6, and the relevant parameters fitted by the two isothermal adsorption models are shown in Table 1. We can see that compared with the correlation coefficients fitted by the Freundlich model, the correlation coefficients fitted by the Langmuir isothermal adsorption model of composite hydrogel materials for Pb^2+^, Cu^2+^, and Cd^2+^ are 0.9951, 0.9901, and 0.9973, respectively, which were closer to 1. This indicates that the adsorption process of heavy metal ions by the hydrogel can be fitted using the Langmuir model, which also suggests that the adsorption process is primarily dominated by monolayer chemisorption. The Langmuir model further indicates that the majority of Pb^2+^, Cu^2+^, and Cd^2+^ ions are adsorbed on the active adsorption sites of the CD/CMC-PAA-MBA surface. The Langmuir model fitting reveals the maximum adsorption capacities of Pb^2+^, Cu^2+^, and Cd^2+^ by the hydrogel to be 182.48 mg/g, 636.94 mg/g, and 290.69 mg/g, respectively. From the relevant data fitted by the Freundlich model, it can be seen that the value of n, which was the characteristic constant for the adsorption of Pb^2+^, Cu^2+^, and Cd^2+^ by the hydrogel, was 3.39, 1.86, and 2.55, respectively, and all of them were greater than 1, indicating that the adsorption process is occurs easily and belonged to preferential adsorption. In the Temkin isotherm adsorption model, a larger value of B indicates a more significant variation of adsorption heat with the coverage of the adsorbent and a faster decrease in adsorption heat during the adsorption process. Consequently, we observed the fastest variation in adsorption heat when the adsorbent adsorbs Pb^2+^.

#### 2.2.6. Adsorption Kinetics

To elucidate the impact of CD/CMC-PAA-MBA on adsorption kinetics, experimental data were analyzed using a kinetic model. This model primarily depicts the adsorption process as a function of time, reflecting the transition curve of adsorption quantity over the adsorption duration. The observed curves elucidate the distribution dynamics of the adsorbate between the adsorbent and solution phases. This study focused on elucidating the adsorption mechanism of the synthesized composite hydrogel through the application of two kinetic adsorption models: the pseudo-first-order and pseudo-second-order models. The pseudo-first-order model postulates that the adsorption process is governed predominantly by physical diffusion, as described by Equation (3). In contrast, the pseudo-second-order model posits that adsorption is influenced by electron sharing, exchange, and transfer mechanisms, essentially involving the formation of covalent bonds, as represented by Equation (4) [35].

(4) $$\log\left(Q_{e}-Q_{t}\right)=logQ_{e}-\frac{k_{1}}{2.303}t$$

(5) $$\frac{t}{Q_{t}}=\frac{1}{k_{2}Q_{e}^{2}}+\frac{t}{Q_{e}}$$

In the equation, Q_t_ is the amount adsorbed after time t, in mg/g; Q_e_ is the amount adsorbed after adsorption equilibrium of the adsorbent, in mg/g; k_1_ and k_2_ are the pseudo-first and pseudo-second order kinetic rate constants; t is the contact time between the adsorbent and the sorbate.

**Figure 7:**
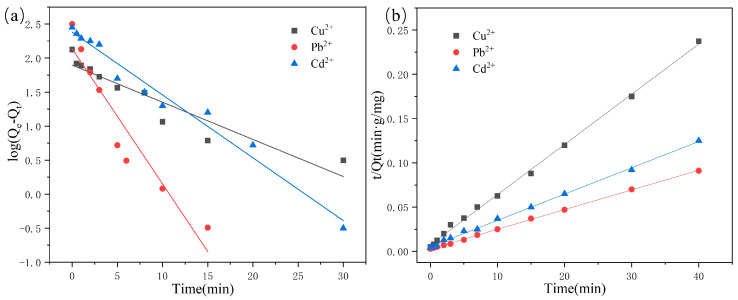
(**a**) Pseudo−first−order and (**b**) pseudo−second order kinetic adsorption model for Pb^2+^, Cd^2+^, and Cu^2+^ adsorption.

The fitting curves and parameters derived from the pseudo-first-order and pseudo-second-order kinetics models, as presented in Figure 7a,b, and Table 2, demonstrate significant discrepancies between the theoretical (Q_e_) values of the pseudo-first-order model and the experimental data. In contrast, the theoretical (Q_e_) values from the pseudo-second-order model align closely with the experimental results. Moreover, the pseudo-second-order model exhibits a higher correlation coefficient (R^2^), approaching unity, compared to the pseudo-first-order model. Consequently, these findings indicate that the adsorption of the three metal ions by the composite hydrogel is accurately simulated by the pseudo-second-order kinetic model, underscoring that the adsorption process is predominantly chemisorption [36].

#### 2.2.7. Regenerative Performance of Prepared Hydrogels

**Figure 8:**
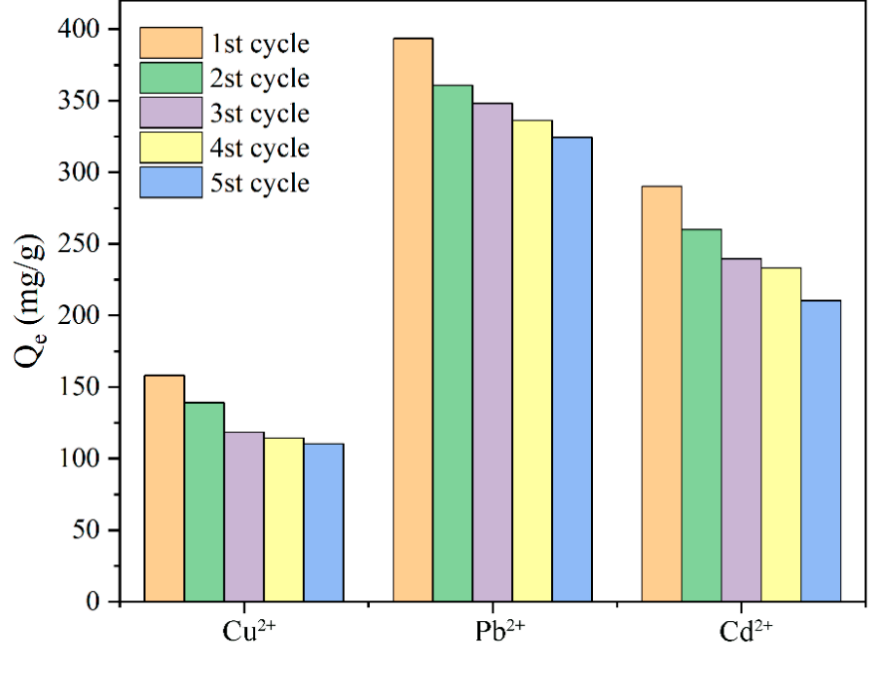
Experiments of CD/CMC−PAA−MBA regeneration performance.

**Figure 9:**
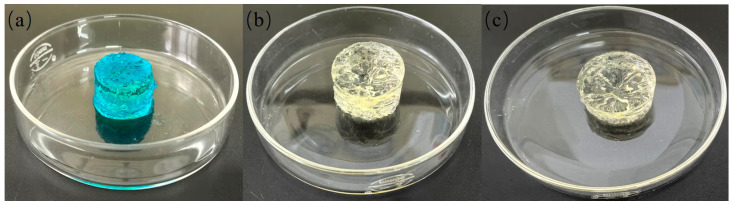
(**a**) Digital photo of the form of Cu^2+^ adsorbed by hydrogel, (**b**) digital photo of the form of Pb^2+^ adsorbed by hydrogel, and (**c**) digital photo of the form of Cd^2+^ adsorbed by hydrogel.

The regenerative performance of hydrogel adsorbents is a crucial parameter for assessing their potential for recycling. The development of hydrogels with outstanding regenerative capabilities is not only an urgent challenge for current researchers but also aligns with the pressing needs of modern sustainable development. This study examined the regeneration performance of the CD/CMC–PAA–MBA composite hydrogel through five cycles of desorption–adsorption experiments, as depicted in Figure 8. The results indicated that even after five cycles, the hydrogel maintained a high adsorption capacity, demonstrating excellent repeatability [37]. The cyclic morphology of hydrogel after adsorption of heavy metals is shown in Figure 9.

#### 2.2.8. Competitive Adsorption of Metal Ions

**Figure 10:**
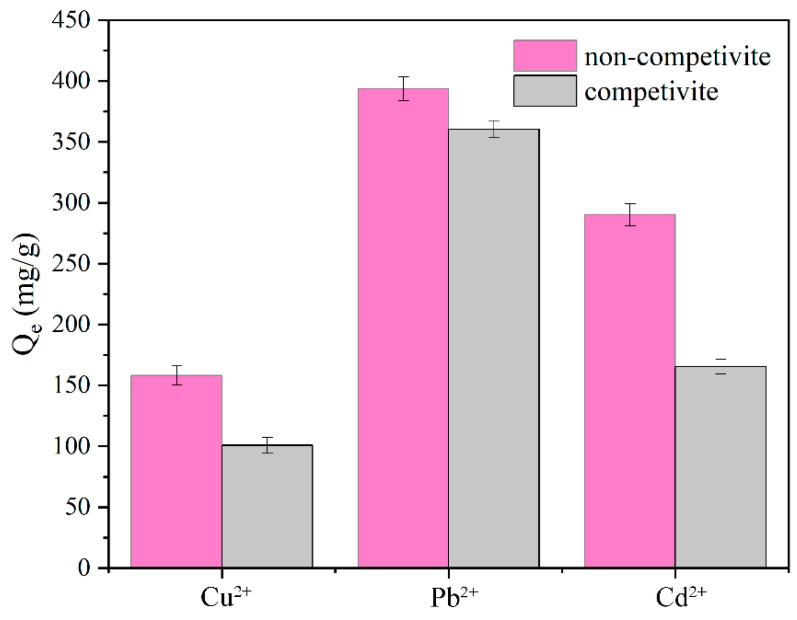
Adsorption of metal ions on CD/CMC−PAA−MBA composite hydrogels.

Various heavy metal ions including Cu^2+^, Pb^2+^, and Cd^2+^ are frequently used and found in industrial wastewater. Therefore, in this study, individual and mixed solutions containing Cu^2+^, Pb^2+^, and Cd^2+^ metal ions were prepared to investigate the selective adsorption of metal ions on CD/CMC–PAA–MBA composite hydrogels. To adsorb specific metal ions, the hydrogels were immersed in individual metal ion solutions. The results showed that the adsorption capacity of the hydrogel for Pb^2+^ was about 393.56 mg/g, whereas the adsorption capacity for Cu^2+^ and Cd^2+^ was 158.12 and 290.12 mg/g, respectively (Figure 10). Compared with other divalent metal ions, the hydrogel surface may contain more groups that can form stable complexes with Pb^2+^, such as hydroxyl (-OH), carboxyl (-COOH), etc., which have a stronger coordination capacity with Pb^2+^ than Cu^2+^ and Cd^2+^. It is known that the amount of adsorption is affected by the size of the metal ions, and the pore structure and pore size distribution of the hydrogel may be more suitable for the diffusion and adsorption of Pb^2+^, making it easier for Pb^2+^ to enter and be immobilized in the pores [38].

## 3. Materials and Methods

### 3.1. Materials and Instruments

*β*–cyclodextrin (CD, A.R.) and carboxymethyl cellulose (M.W. 90,000, 50–100 mPa.s) were obtained from Shanghai Aladdin Biochemical Technology Co., Ltd. (Shanghai, China). Acrylic acid (AA), ammonium persulfate (APS), and *N*, *N*-Methylenebisacrylamide (MBA) were purchased from J&K Scientific Co., Ltd. (Beijing, China). Sodium hydroxide (NaOH, A.R.), hydrochloric acid (HCl, A.R.), copper chloride (CuCl_2_, A.R.), lead nitrate (PbNO_3_, A.R.), and chromic chloride (CdCl_2_, A.R.) were obtained from National Pharmaceutical Group Chemical Reagent Co., Ltd. (Shanghai, China). All reagents used were as received, not purified, and all solutions used were prepared with deionized water.

In this study, the following equipment was used in the experimental process: an atomic absorption spectrophotometer (Shimadzu Instruments Japan Ltd., Kyoto, Japan, model: AA AA 6300), laboratory analytical balance (Sartorius Lab Instruments GmbH £t Co. KG, Goettingen, Germany, model: GL1241-1SCN), collecting magnetic stirrer with constant temperature heating (Gongyi City Zihua instrument Co., Ltd., Zhengzhou, China, model: DF-101S), lyophilizer (*BUCHI* Labortechnik AG, model: BUCHI-L200), pH meter (Mettler Toledo Instruments Co., Ltd., Shanghai, China, model: S220), electrothermal blast drying oven (Shanghai Boxun Industry Co., Shanghai, China, model: GZX-9070MBE), and UV spectrophotometer (Hitachi (China) Ltd., Beijing, China, model: U-3900).

### 3.2. Preparation of CD/CMC-PAA-MBA Hydrogel Materials

First, 1 g of CMC powder was dissolved in 50 mL of deionized water in a 250 mL three-necked flask equipped with a mechanical stirrer, reflux condenser, and nitrogen tube, sonicated and stirred to form a clear viscous solution, and 0.5 g of β-cyclodextrin powder was added with continued stirring until completely dispersed. The solution was heated to 70 °C, and nitrogen was introduced for 20 min to completely remove oxygen from the solution. Then, 8.2 g, MW% = 70% of AA monomer was added, 0.2 g of MBA cross-linker was added, and the mixed solution was mechanically stirred for about 1 h until complete dissolution while keeping the temperature at 70 °C constant and nitrogen gas was passed through. After cooling to room temperature, the initiator APS (MW% = 5%, 5 mL) was added, stirred well, poured into the mold, and placed in the oven to be heated to 70 °C for 30 min to become a hydrogel, named CD/CMC-PAA-MBA.

### 3.3. Characterization

All the prepared hydrogel samples were cold dried with a BUCHI-L200 lyophilizer for subsequent testing. The functional groups of the hydrogel samples were characterized via Fourier transform infrared spectroscopy ATR (PerkinElmer FTIR Spectrometer Spectrum Two, Shelton, CT, USA) in the scanning range of 4000–400 cm^−1^ with a resolution of 4 cm^−1^. The crystal structures were characterized through powder X-ray diffraction (XRD) analysis, utilizing a Rigaku Smart Lab X-ray diffractometer (Rigaku, Tokyo, Japan) at 5–80°. The fracture surface morphologies were examined using a JEOL-JSM-6700F scanning electron microscope (SEM) (JEOL Company, Tokyo, Japan) with an accelerated voltage of 5.0 kV. A simultaneous thermal analyzer (HITACHI STA200, Tokyo, Japan) measured the weight change of the hydrogel samples under heating conditions, with a heating rate of 10 °C/min from room temperature to 500 °C in a (N_2_, Air, Ar) atmosphere. The compression resistance of the hydrogel samples was tested using a universal testing machine (Jilin Guanteng Automation WDW-1, Changchun, China) at a testing rate of 1 mm/min at room temperature. The viscoelastic properties of the hydrogel samples were tested using a rheometer (Anton Paar MCR92, Graz, Austria).

### 3.4. Adsorption Experiments

The initial mass concentration of heavy metal ions and pH were taken as variables, respectively, and other conditions were kept consistent to investigate the effects of different factor conditions on the adsorption of heavy metal ions on hydrogels.

In the isothermal adsorption experiments, the initial concentrations of Cu^2+^ and Pb^2+^ solutions were set at 50–500 mg/L and the initial concentrations of Cd^2+^ were set at 50–2000 mg/L.

The adsorption experiments were carried out at a constant temperature of 25 °C and 150 rpm on a water bath shaker: 15 mL of a certain mass concentration of CuCl_2_ solution, PbNO_3_ solution, CdCl_2_ solution, and 10 mg of hydrogel samples were added into a 100 mL conical flask sequentially and then reacted for several hours in the water bath shaker until the adsorption reached the equilibrium, and the supernatant in the flask was sucked up to quantitatively determine and dilute the solution. The solution was quantitatively determined and diluted. The concentrations of heavy metal ions Cu^2+^, Pb^2+^, and Cd^2+^ in the measured supernatant were determined via Shimadzu AA-6880 atomic absorption spectrometry (flame method). The adsorption capacity of the hydrogel for Cu^2+^, Pb^2^, and Cd^2+^ was calculated according to Equations (1) and (2), respectively [39].

(6) $$q_{e}=\frac{(C_{0}-C_{e})V}{m}$$

(7) $$q_{t}=\frac{(C_{0}-C_{t})V}{m}$$

q_t_ (mg/g) is the adsorbed amount of hydrogel at moment t; q_e_ (mg/g) is the adsorbed amount of hydrogel when the adsorption reaches equilibrium; C_o_ (mg/L) is the initial concentration of Cu^2+^, Pb^2+^, and Cd^2+^; C_e_ (mg/L) is the Cu^2+^, Pb^2+^, and Cd^2+^ solutions at the time the adsorption reaches equilibrium; C_t_ (mg/L) is the concentration of Cu^2+^, Pb^2+^, and Cd^2+^ solutions at the moment t; V (L) is the volume of the metal ion solutions; m (mg) is the mass of the substance.

### 3.5. Reusability Test of CD/CMA-PAA-MBA Hydrogel

In order to realize the reusability of the hydrogel materials, the hydrogel materials that had completed the adsorption experiments were placed in a 1 M hydrochloric acid solution for metal ion desorption, and the reaction time was 1 h. They were washed five times repeatedly with deionized water until the pH reached neutrality. Subsequently, the hydrogel material was lyophilized, weighed, and used in the next round of adsorption experiments [36]. The above adsorption experiments were repeated five times to examine the regenerative properties of the hydrogel materials.

## 4. Conclusions

In this study, we synthesized an environmentally friendly CD/CMC-PAA-MBA composite hydrogel through radical polymerization cross-linking, showcasing superior mechanical properties and an elevated recycling efficiency. This hydrogel retains the essential frameworks of CD and CMC and was utilized for the adsorption of Cu^2+^, Pb^22^, and Cd^2+^ from wastewater. Characterization via FTIR-ATR, XRD, TGA, and SEM confirmed the successful grafting of AA and MBA monomers onto the CD and CMC backbones, creating a dual-network structure. The newly synthesized hydrogels exhibit a coarse, irregular, and porous surface, coupled with excellent thermal stability. We investigated the effects of four key parameters—pH, temperature, contact time, and initial concentration—on the adsorption of metal ions by the hydrogel. Results indicated that under optimal conditions—a pH of 4, a temperature of 25 °C, a contact time of 15 min, and an initial concentration of 400 mg/L—the maximum adsorption capacities were 158.12 mg/g for Cu^2+^, 393.56 mg/g for Pb^2+^, and 290.12 mg/g for Cd^2+^, respectively. Using isothermal adsorption models and kinetic adsorption models, we elucidated the mechanism by which the composite hydrogel adsorbs metal ions. Notably, after undergoing five cycles, the hydrogel maintained a relatively high adsorption capacity, demonstrating its excellent cyclic stability and robust mechanical properties. These findings suggest that the composite hydrogel is well-suited for the efficient treatment of metal ion-contaminated wastewater.

## Figures and Tables

**Table 1:** Adsorption isotherm parameters for Cu^2+^, Pb^2+^, and Cd^2+^.

	Langmuir			Freundlich		
	Q_m_	K_L_	R^2^	n	K_p_	R^2^
Cu^2+^	182.48	0.1034	0.9951	3.39	10.08	0.8005
Pb^2+^	636.94	0.0985	0.9901	1.86	9.17	0.3950
Cd^2+^	290.69	0.0867	0.9973	2.55	10.55	0.7474
	**Temkin**					
	**B**		**K_T_**		**R^2^**	
Cu^2+^	34.61		1.22		0.8841	
Pb^2+^	162.95		0.24		0.6184	
Cd^2+^	46.41		3.31		0.7071	

**Table 2:** Kinetic adsorption model parameters for Cu^2+^, Pb^2+^, and Cd^2+^.

	Pseudo-First-Order			Pseudo-Second-Order		
	K_1_	Q_e1_	R^2^	K_2_	Q_e2_	R^2^
Cu^2+^	0.1257	78.83	0.8889	0.0043	176.37	0.9984
Pb^2+^	0.4575	137.04	0.9199	0.0018	450.45	0.9996
Cd^2+^	0.2126	240.65	0.9752	0.0017	336.70	0.9979

## Data Availability

The data presented in this study are available on request from the corresponding author share their research data.
